# Challenges and Resiliency: Social Determinants of Health, COVID-19, and the Disproportionate Impact on Immigrants and Refugees Living with HIV

**DOI:** 10.3390/ijerph22010114

**Published:** 2025-01-15

**Authors:** Natasha Marriette, Rita Dhungel, Karun Kishor Karki, Jose Benito Tovillo

**Affiliations:** 1 School of Social Work and Human Services, University of the Fraser Valley, Abbotsford, BC V2S 7M8, Canada; rita.dhungel@ufv.ca; 2 School of Social Work, University of British Columbia, Vancouver, BC V6T 1Z2, Canada; karun.karki@ubc.ca; 3 Faculty of Nursing, MacEwan University, Edmonton, AB T5J 4S2, Canada; tovilloj@macewan.ca

**Keywords:** HIV, social determinants of health, COVID-19, employment status, immigrants and refugees

## Abstract

The human immunodeficiency virus (HIV) pandemic is a global public health and social justice issue. HIV continues to disproportionately affect marginalized populations, including immigrants and refugees living with HIV (IRLHIV). This study investigated and captured the experiences of IRLHIV using the social determinants of health framework. This study examined the intersecting factors affecting the health and well-being of IRLHIV in Alberta, Canada, prior to and during the COVID-19 pandemic. Concurrent mixed methods were used. Employing an online survey (n = 124) and photovoice methodology (n = 13), the researchers identified five salient themes: experiences of racism and discrimination, challenges accessing nutrition, healthcare, and affordable housing, and precarious employment situations. The findings underscored the amplification of pre-existing inequities during the COVID-19 pandemic, intensifying the discrimination and stigma faced by IRLHIV due to both their health status and immigration background. These findings highlight the urgent need for targeted, evidence-based interventions to address the social determinants of health that adversely affect IRLHIV. The researchers recommend further participatory research action into health disparities for IRLHIV to create responsive and culturally safe services for IRLHIV.

## 1. Introduction

Human immunodeficiency virus (HIV) is a virus that attacks the body’s immune system, and when left untreated, it can lead to acquired immunodeficiency syndrome (AIDS) [1]. HIV/AIDS is a worldwide pandemic with an estimated number of 39.9 million people living with HIV in 2023 [2]. Since the beginning of the HIV/AIDS pandemic, approximately 88.4 million people globally have been diagnosed with HIV [2]. In 2023, 1.3 million people were newly identified as living with HIV while 650,000 people died from illnesses related to AIDS [2]. In 2022 there were 1833 first-time diagnoses of HIV in Canada [3]. This is an increase of 24.9% compared with the number of cases reported in 2021 (1468 cases). Of the 1833 diagnoses, 190 were in the province of Alberta. This is an increase from the 179 first-time diagnoses in Alberta in 2021 [3].

Prior to COVID-19, Canada introduced a pan-Canadian sexually transmitted and blood-borne infections (STBBI) framework [4]. This framework was in place to address STBBI which includes HIV, hepatitis B and C, chlamydia, and other STBBI [4]. The framework was put in place with four main goals: prevention, testing, care and treatment, and ongoing care. The Government of Canada identified newcomers and immigrants to Canada as key populations affected by STBBI [5]. While the STBBI framework includes services for these populations, the Government of Canada [5] also states that factors such as social determinants of health can affect the rates of STBBI. According to the Government of Canada [5], barriers affecting the reduction in rates of HIV include some individuals having little to no symptoms when the disease is first transmitted and the fear of judgment if an individual were to be tested for HIV. HIV remains a global social justice and public health issue, claiming 40.1 million lives through ongoing transmission across the globe [1].

At the end of 2022, 65,270 individuals in Canada were living with HIV/AIDS [3]. There was an overall increase in newly diagnosed cases of HIV of 24.9% in Canada. British Columbia (BC) had an increase of 2.5% in newly diagnosed cases; Ontario had a 4.1% increase, and Alberta had a 4.2 percent increase [6]. While HIV is not curable, timely diagnosis, treatment, and care allow HIV to be managed as a chronic health condition [1]. Despite the absence of a cure for HIV, antiretroviral therapy can effectively prevent its progression to AIDS and prevent sexual transmission [2]. UNAIDS has a 95–95–95 target for 2025. This means that 95% of people living with HIV know their HIV status; 95% of people living with HIV who know they are HIV positive are on antiretroviral therapy, and 95% of those on antiretroviral therapy have suppressed viral loads.

HIV disproportionately affects populations such as individuals who use injectable substances, ethnic and cultural minorities, LGBTQ2S+ populations, and incarcerated individuals [7]. Despite the availability of prevention strategies, diagnosis, treatment, and care for HIV and AIDS, inequitable access persists [5]. This inequity disproportionately affects marginalized communities, including immigrants and refugees, who encounter distinctive barriers in accessing essential HIV/AIDS services in Canada [8]. One issue is unclear and results in incomplete data on the rates of HIV/AIDS among immigrants and refugees in Canada [9]. Statistics Canada does not collect or disseminate the number of people affected by HIV; this information is dependent on the results of Immigration Medical Exam (IME) [8]. Any person over the age of 15 applying for permanent residence is required to have an IME in Canada or overseas as part of their entry to Canada [8]. In 2002, Canada integrated HIV antibody screening into the IME [10]. Between 2013 and 2022, 9208 individuals who received an IME were diagnosed with HIV [3]. A total of 468 of these individuals received their IME in Alberta, making the rate of diagnosis 4.2 per 100,000 people [3].

While immigrants and refugees are vital to Canada’s growth, they face barriers in accessing HIV/AIDS prevention and care [11]. This is partly due to fears associated with an HIV diagnosis, such as potential impacts on immigration status [12,13]. Stigma and misinformation within their communities also play a role [12]. COVID-19 has further complicated the lives of people living with HIV/AIDS (PLWH), especially within the immigrant and refugee populations [9]. The pandemic has heightened feelings of isolation and disrupted access to support services, particularly in Alberta [9,14]. These concerns were felt exponentially by immigrants and refugees living with HIV (IRLHIV), as lockdowns and health fears can limit access to vital care [9].

Research on culturally appropriate, post-COVID-19 interventions for IRLHIV in Canada is limited. This lack of guidance leaves communities and service providers ill equipped to provide optimal support. Beyond generating knowledge, this study aims to facilitate social transformation by influencing policy and practice, ultimately improving the lives of IRLHIV in Canada. Culturally appropriate services are essential not only for addressing healthcare needs but also for fostering overall well-being. By prioritizing culturally appropriate care, this study endeavors to investigate and document the experiences of IRLHIV to improve the health and quality of life of IRLHIV in Canada.

This study is grounded in the Social Determinants of Health framework for the purpose of examining the intersecting factors experienced by IRLHIV in Alberta, Canada prior to and during the COVID-19 pandemic. How do IRLHIV in Alberta experience Social Determinants of Health and health inequities in their lives?

## 2. Theoretical Framework

### Social Determinants of Health (SDH)

**Figure 1:**
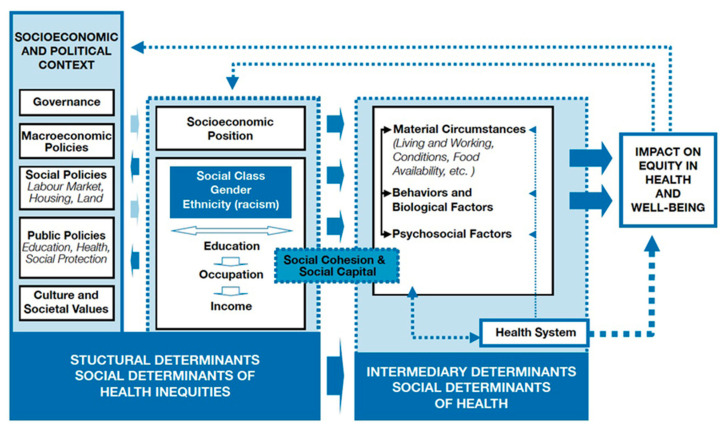
Commission on the social determinants of health conceptual framework. Note. Figure 1 was retrieved from the WHO’s publication of A Conceptual Framework for Action on the Social Determinants of Health (2010) and included in this article as per the CC BY-NC-SA 3.0 IGO license [19].

This study employed the SDH framework, which was derived from the Whitehall studies [15]. These studies demonstrated an inverse correlation between employment level and mortality, highlighting the impact of social factors like work environment and social hierarchy on health outcomes. The World Health Organization defines SDH as “non-medical factors that influence health outcomes”, encompassing both physiological and psychological well-being. Understanding SDH allows for the development of holistic healthcare approaches that address root causes and consider the intersectionality of various social factors [16]. SDH encompasses an individual’s living and working conditions, social environments, and broader economic, political, and social factors that affect health and well-being [17,18]. These include social inclusion, geography, colonization, working conditions, education, and structural conflict. Figure 1 provides a visual of the SDH framework by the World Health Organization [19]. The SDH framework was chosen as it is a widely known and developed framework, particularly in public health [17]. The SDH framework crosses interdisciplinary boundaries and provides an actionable lens for understanding and responding to health disparities, including communicable and non-communicable diseases [17].

Social inequities and socioeconomic status contribute to health disparities, even in affluent countries like Canada [18]. The COVID-19 pandemic exacerbated these inequalities, further disadvantaging those already experiencing social and economic disparities [18]. This study investigates the lived experiences of immigrants and refugees living with IRLHIV in Alberta, Canada. It focuses on IRLHIV’s experiences of SDH and health inequities. By examining these lived experiences, this research aims to provide recommendations for culturally appropriate supports, services, and policies to address health inequities faced by IRLHIV in Alberta.

## 3. Methods

### Methodological Approach

This study was completed in Alberta, Canada, and used the concurrent mixed-methods approach [20]. The quantitative portion of the study employed a cross-sectional online survey to immigrant and refugee populations in Alberta, Canada. Populations that are marginalized or underserved can be difficult for researchers to access [21]. This was a particular concern with IRLHIV populations. Snowball sampling was chosen for its effectiveness in reaching hidden or hard-to-reach populations [22]. A consideration in the use of snowball sampling was the potential bias and over-representation due to social connections [21,22]. However, snowball sampling has been shown to be effective for studies with multiple eligibility requirements [21], and sampling can also contribute to cultural safety [21], which was an important consideration for this study. A community connector with knowledge of IRLHIV populations supported the recruitment of participants and the administration of the survey.

The survey was completed from September to December 2021. Participants were 18 years or older and represented diverse demographics, geographic categories, sexual orientations, gender, age, education, and more. HIV status was self-reported. The study and invitations for participants were advertised in community venues, social events, and posted on relevant internet forums, web pages, and social media sites. A total of 124 participants’ responses were captured with a completion rate of 82%. The survey collected demographic information of participants, explored the experiences of IRLHIV, the impacts of mental health and psychological well-being prior to and during the COVID-19 pandemic, and experiences related to SDH. Table 1 below displays the sociodemographic characteristics of the survey participants.

Participants were informed of the purpose of the study. Information for crisis intervention services were included in the introduction of the survey for individuals who felt distress, anxiety, or any mental health concerns during or after the survey. Participants were informed of their right to withdraw consent from the survey at any time. Survey data were kept confidential and only viewed by members of the research team.

The study employed a participatory action approach with a photovoice methodology for its qualitative component. Photovoice is a participatory research method that empowers individuals to express their perspectives on community issues through photography and dialog, using visual storytelling to influence policy and decision-making [23]. Photovoice creates opportunities for those who are marginalized and whose voices are silenced, allowing them to actively participate in enhancing their communities by giving them a chance to tell their stories and have their voices heard [23,24]. Participants are equipped with cameras so that they can create photographic evidence and symbolic representations to help others see the world through their eyes [24,25].

Photovoice participants are co-researchers and co-creators of knowledge, and therefore, it is important for them to have a great understanding of how photovoice projects could be conducted in fair, ethical, and appropriate ways. The participants were educated on the research process of informed consent and on the process of operating in political arenas [26]. This training included participants’ own safety and well-being. A total of 34 photovoices were created by 13 participants from September to December 2021. Participants were from across Alberta. These individuals were identified as IRLHIV. To make the study accessible and affordable to all, participants were offered disposable cameras if required.

Photovoice participants were informed of their right to withdraw consent at any time. Crisis intervention resources were offered to participants in case they felt any distress or emotional concerns before, during, or after the photovoice process. The consent form included permission for data to be presented in journals and conferences.

Ethics approval was received from the MacEwan University ethics review committee in Edmonton, Alberta. The ethics approval number is 101907.

To analyze the photovoice data, we adopted a comprehensive and collaborative approach grounded in thematic analysis, as outlined by [27]. Their methodology allowed us to systematically identify, analyze, and report patterns within the data. Our process involved a series of interconnected stages, each crucial to ensure the rigor and validity of our findings. Initially, each co-author independently immersed themselves in the visual narratives presented within the photographs. This familiarization phase involved careful observation and reflection, allowing each researcher to develop an individual understanding of the participants’ experiences and perspectives. This individual engagement with the data was essential for minimizing initial bias and ensuring that diverse interpretations were considered.

Following this, we individually engaged in initial coding, a process of systematically identifying and labeling significant features within the data. This involved scrutinizing the photographs for recurring themes, ideas, and emotions conveyed by the participants. This stage marked the beginning of our collaborative analysis, as we transitioned from individual interpretations to a shared understanding of the data. We then moved into a more interpretive phase, where we independently developed initial themes and subthemes based on the codes generated in the previous stage. This involved identifying connections and relationships between codes, grouping them into broader categories that captured the essence of the participants’ experiences. This individual theme development allowed for a variety of perspectives to emerge, enriching the subsequent collaborative discussions.

The core of our analysis resided in the bi-weekly virtual meetings held over five months. These meetings served as a platform for in-depth discussions and deliberations, where we critically examined each other’s proposed themes and subthemes. This iterative process of refinement involved challenging assumptions, clarifying ambiguities, and negotiating meanings to ensure that the final themes accurately and comprehensively reflected the data. Through these collaborative discussions, we gradually moved towards consensus, carefully defining and naming the overarching themes that formed the backbone of our analysis. This process ensured that the final thematic framework was robust, nuanced, and grounded in the shared understanding of the research team.

The result of this process is presented in the next section of this paper, where we provide a detailed discussion of the final themes. It provides a comprehensive account of our findings, offering valuable insights into the lived experiences of IRLHIV during the COVID-19 pandemic.

## 4. Results and Discussion

### 4.1. Themes

The following five SDH emerged in our analysis of completed surveys and photovoices: (1) racism and discrimination, (2) access to nutrition, (3) access to healthcare, (4) access to affordable housing, and (5) employment status. Quantitative and qualitative findings will be discussed by theme, with qualitative results including the use of photovoices.

### 4.2. Racism and Discrimination

**Figure 2:**
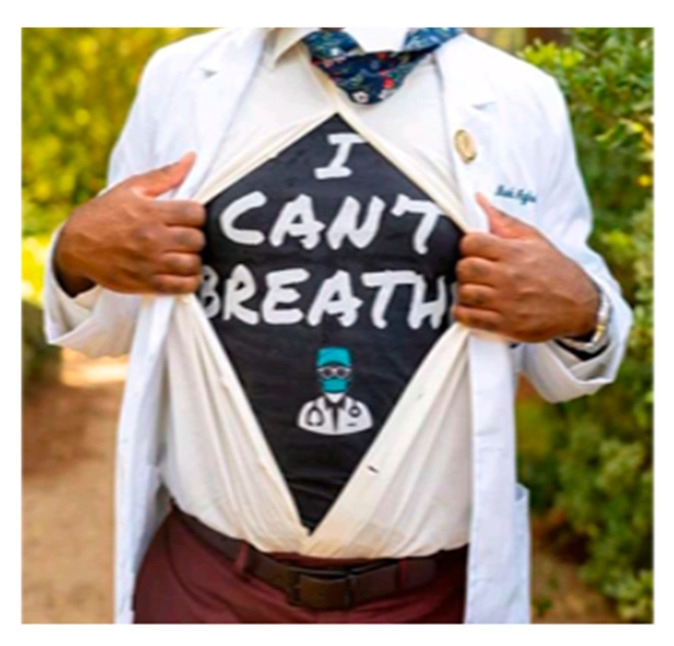
“This picture I took it after being discriminated just because I look different. We are treated differently because of our colour. I know others living with HIV do not have to go through the same experience as we do everywhere.”

**Figure 3:**
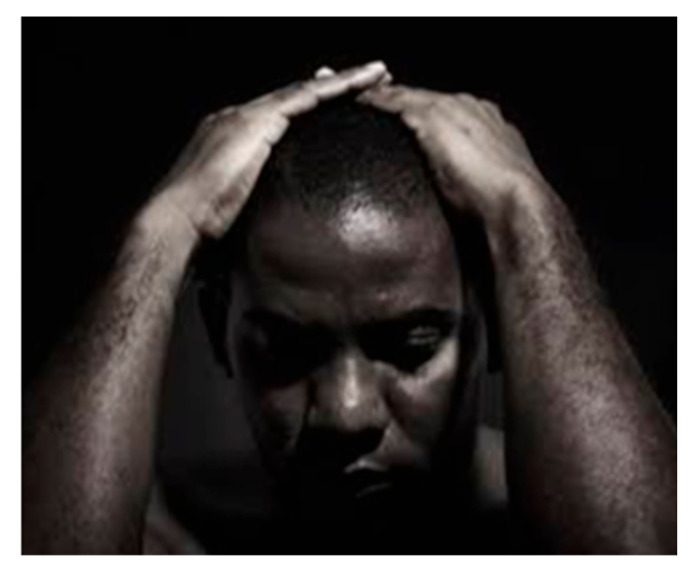
“It’s very difficult being a person of color living in another country especially low wage country like Canada. Then being a person of color and also a person living with HIV. You can imagine how hard our life would be like we are the outcast like we don’t deserve to be treated fairly.”

The Canadian Race Relations Foundation (CRRF) defines racism as “a belief that one group is superior to others performed through any individual action, or institutional practice which treats people differently because of their colour or ethnicity” [28]. These categories are then used to justify the unequal treatment of certain individuals and groups based on those characteristics. Discrimination is “the denial of equal treatment and opportunity to individuals or groups because of personal characteristics and membership in specific groups” [28]. The survey participants were asked whether the discrimination they experienced had increased since the COVID-19 pandemic started. More than 59% of participants stated that they had experienced discrimination either most of the time (49.1%), some of the time (23.6%), or all of the time (10%). This differential treatment prevents equal opportunity. Findings from the photovoices identified the SDH of racism as negatively affecting the health outcomes of IRLHIV. Figure 2 and Figure 3 are photos shared and narrated by research participants.

Our survey results further corroborate with these findings. Notably, the bivariate correlation presented in Table 2 reveals a significant association between the variables “discrimination” and “stigma” with respect to health services and social support, both before and during the COVID-19 pandemic.

During the COVID-19 pandemic, a robust positive correlation emerged between respondents’ social support levels and their well-being (r(108) = 0.88, *p* = 0.002), indicating a strong relationship. Conversely, a negative correlation was found between perceived stigma levels and well-being (r(106) = 0.330, *p* < 0.001), highlighting the detrimental impact of stigma on individuals’ well-being. Additionally, individuals experienced significant challenges related to social determinants of health, such as childcare and housing, during the pandemic, leading to diminished physical and mental health outcomes. However, increased social support emerged as a protective factor, promoting and safeguarding well-being in the face of these challenges. The theme of access to healthcare resonates with the findings of [29], whose study highlighted that immigrants and refugees face challenges and obstacles in obtaining healthcare services.

#### 4.2.1. Access to Food Security

**Figure 4:**
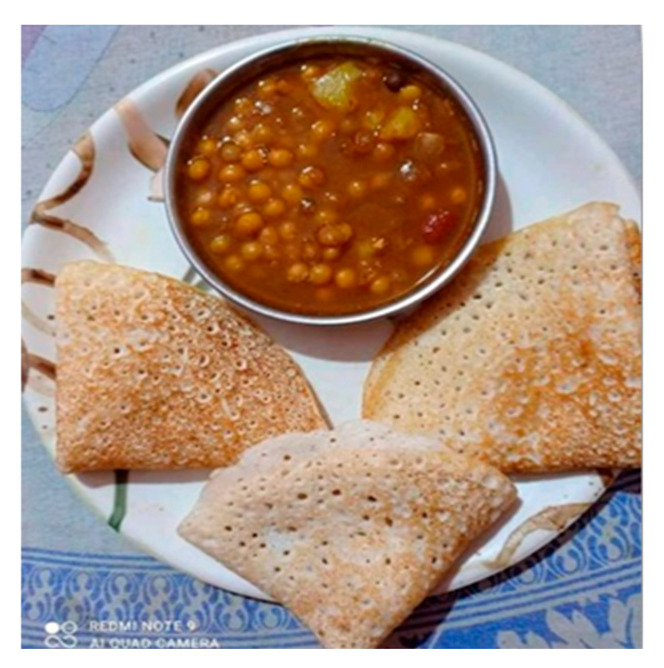
A photo representing a participant’s access to cultural and nutritious food.

**Figure 5:**
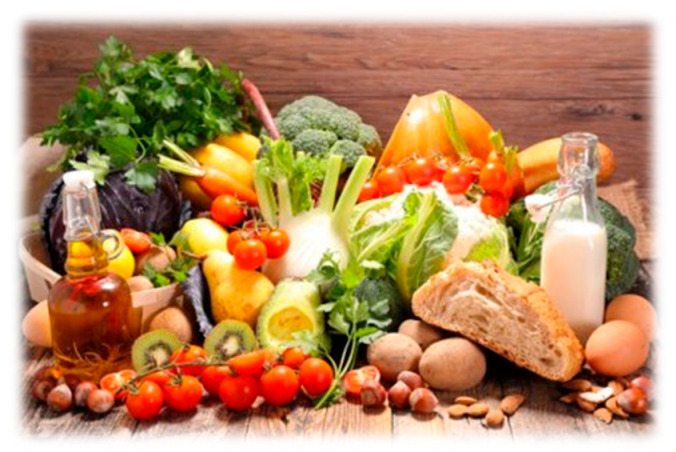
A photo shared and narrated by a research participant to represent food insecurity.

A second theme that emerged from the data was access to food security. Food security is the certainty that an individual can acquire nutritious, culturally appropriate food in socially acceptable ways [18]. Survey participants were asked about their health in general amid the COVID-19 pandemic. As shown in the pie chart, of the 110 participants, 53% stated their health was poor, and 33% of the participants stated that it was fair. More than 40% of the participants had difficulty accessing nutritious food. A total of 30% of the survey participants reported difficulty accessing food hampers prior to COVID-19, while 73% reported difficulty accessing food hampers during COVID-19. Participants also identified a disconnect between repeated recommendations to eat healthily and challenges in finding and affording healthy food. Figure 4 and Figure 5 are photos shared and narrated by research participants to represent food insecurity.

“The food in the picture is that type of food healthy. I love our traditional food. We all are told to make sure you eat healthy food helping to boost your immunity. But how? The Government is required to make food affordable to People Living with HIV.”

“This picture is about healthy eating a balanced diet. I know we need eat healthy food but at the same time it’s expensive especially during COVID-19 food has increased so high. So it’s almost impossible to buy the healthy food. It would be nice to have some kind of discount for the People living with HIV or affordable food.”

Studies conducted by [24,29,30] identified access to food security as a significant challenge faced by IRLHIV.

#### 4.2.2. Access to Healthcare

While access to food security is a significant challenge faced by IRLHIV, access to healthcare is another critical issue identified by the participants. The author of [31] describes healthcare as “integrated health services to meet people’s health needs throughout their lives; addressing the broader determinants of health through multisectoral policy and action; empowering individuals, families and communities to take charge of their own health” (Section 2). The Canadian medical system is publicly funded and referred to as medicare. Provinces and territories share responsibility for the cost and provision of medically necessary healthcare services such as hospital and physician services [32]. Healthcare in Alberta, Canada, is provided by the Alberta Health Services, a province-wide integrated healthcare system [33].

**Figure 6:**
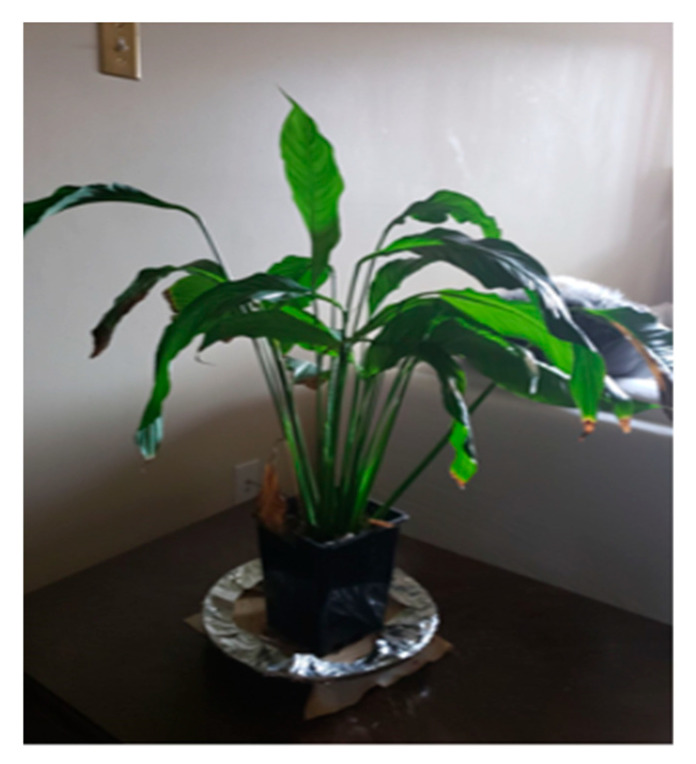
A photo shared and narrated by a research participant portraying the experience of trying to find a family doctor.

**Figure 7:**
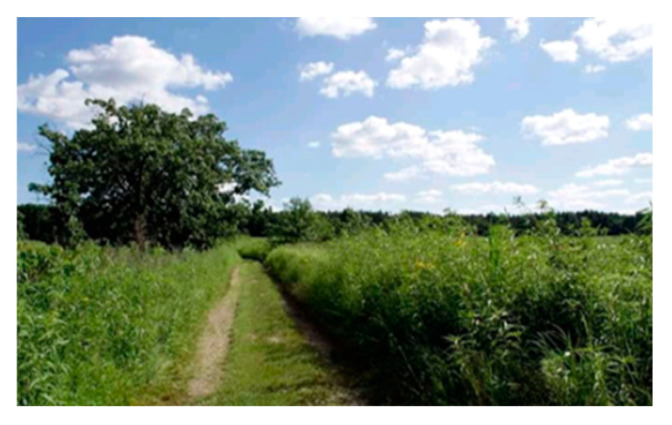
A photo shared and narrated by a research participant portraying the experience of being ignored by healthcare practitioners.

Survey participants and photovoice participants identified challenges in accessing health services prior to and during COVID-19. A total of 22% of the survey participants reported difficulty accessing hospital services prior to COVID-19, and 71% of the survey participants reported difficulty accessing hospital services during COVID-19. Prior to COVID-19, 35% of the survey participants had difficulty accessing health services, while 79% experienced difficulty accessing health services during COVID-19. A total of 23% of the survey participants rated their overall health as fair, while 53% rated it is as poor. Figure 6 and Figure 7 are photovoices narrated by two participants from this study and express difficulty in finding a primary physician due to the intersection of being a person of color and HIV positive.

“The first time when I came here, I was excited and very hopeful looks like this flower. I went to look for a family doctor in NW but that man refused to be my doctor when he found I was a Person Living with HIV. One lady doctor told me no worries I’ll be your doctor. He was accepting others; in a few days, one of my friends went to the same doctor and he took her. I felt so bad.”

“This photo represents how someone is been rejected just because of your color skin. Like you can see there are places which is greener growing well, the owner will water and take care the greener one and ignore the unproductive and [unhealthy one], that’s how doctors ignore “dark skin people” and favor “white people”. This is my experience.”

Research by [12,29,34] also observed similar findings.

#### 4.2.3. Access to Affordable Housing

**Figure 8:**
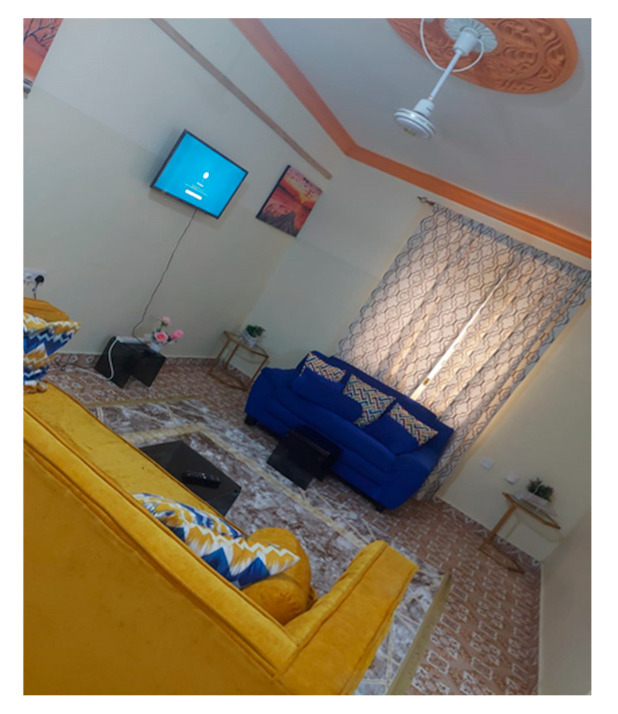
A photo shared and narrated by a research participant portraying the importance of stable housing.

**Figure 9:**
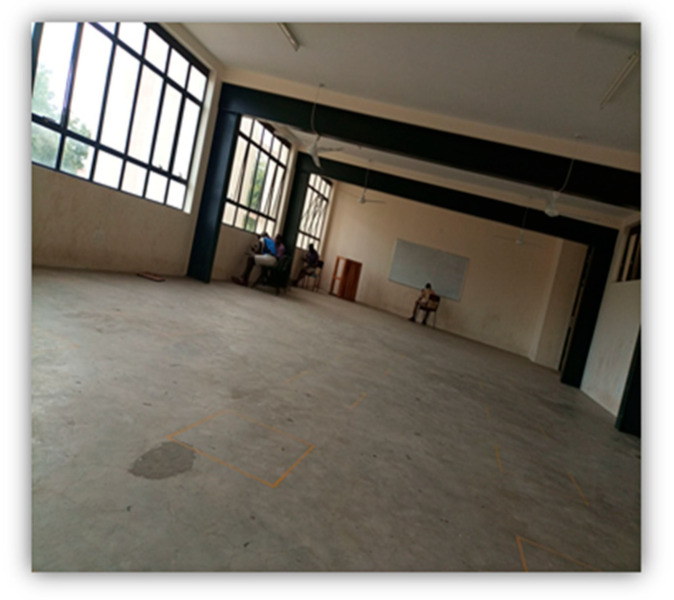
A photo shared and narrated by a research participant portraying their experience regarding the lack of stable housing.

The fourth theme that emerged from the research data was access to affordable housing. The authors of [18] identify affordable housing as “an absolute necessity for living a healthy life and living in unsafe, unaffordable or insecure housing increases the risk of many health problems” (p. 38). Results from the survey found that prior to COVID-19, 55.1% of participants had access to housing services such as low-income housing and eviction support. During COVID-19, that number dropped to 33.7%. A total of 73.7% of participants stated that they agreed or strongly agreed that they experienced increased challenges in maintaining housing during the COVID-19 pandemic. Figure 8 and Figure 9 below further speak to the impact of having housing, a place where IRLHIV could be safe, on their health.

“Yes, this is the place I live in. I just love being in my own space. Basically this picture it’s just my safe zone. The only other thing I’d love if the government could offer better housing services. Like affordable housing services especially for the vulnerable community, like ourselves so we do not have to worry about safety.”

“What is Government doing about housing-nothing? I wish I had a house- house to live at peace housing is so expensive these days and government does not have any plans related to subsidizing/affordable housing for people living with HIV. Having your own house regardless of the size is very important for our mental health-but how?”

The theme of affordable housing reflects findings from [29,30], who identified housing as a bio–psycho–social factor affecting health and well-being. A report completed by [35] found that 62% of newcomers in Edmonton, Alberta, were spending over half their income on rent or mortgage, and housing insecurity caused food instability. Participants in the study completed by [35] also identified long wait times for subsidized housing.

#### 4.2.4. Employment Status

Another theme identified by participants was employment status. “Employment provides income, a sense of identity, and helps to structure day-to-day life. Unemployment frequently leads to material and social deprivation, psychological stress, and the adoption of health-threatening coping behaviours” (18–24). For portions of the COVID-19 pandemic, the Canadian government provided certain financial benefits to offset financial hardships during the pandemic [36]. The Canadian Emergency Response Benefit (CERB) provided $2000 for a four-week period to employed and self-employed Canadians who were unable to work or experienced a reduction in work hours due to COVID-19 [36]. The Canada Recovery Benefit (CRB) was a two-week financial benefit of either $1000 or $600 between 27 September 2020 and 23 October 2021. The Canada Recovery Sickness Benefit (CRSB) provided financial benefits to employed and self-employed individuals who were unable to work or needed to self-isolate due to COVID-19. The Canada Worker Lockdown Benefit (CWLB) provided financial benefits to employed and self-employed individuals who could not work due to the COVID-19 lockdown [36]. These individuals needed to reside in designated lockdown regions. CERB, CRB, CRSB, and CWLB were short term benefits, available to individuals 15 and older and generally required active social insurance numbers (SINs). Eligibility for the above benefits required the individuals to have a certain amount of income in the year(s) prior to applying for the benefits [36].

**Figure 10:**
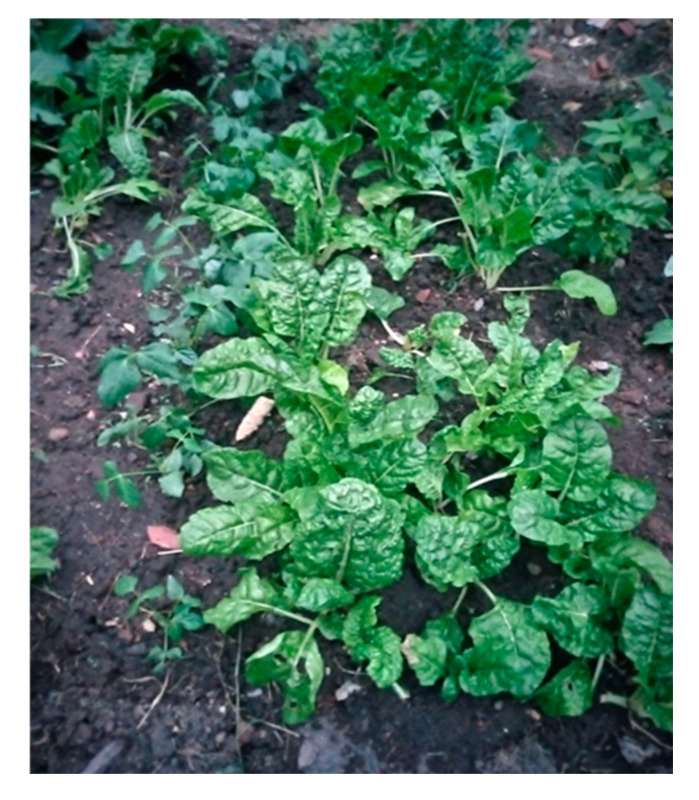
A photo shared and narrated by a research participant expressing resiliency following job loess.

**Figure 11:**
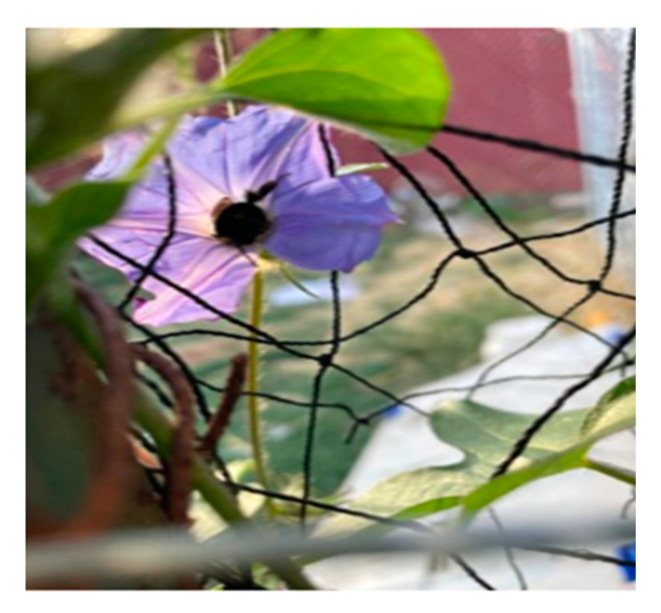
A photo shared and narrated by a research participant about their struggles amidst losing their job.

In the survey, of the 124 participants, more than 26% were unemployed. Only 18% were working full-time, and the majority (55%) of them were working either part-time, casually, or self-employed. The survey findings were corroborated by photovoice findings which portrayed the financial hardships amid the COVID-19 pandemic. Figure 10 and Figure 11 are photovoices completed by participants to describe

“This is a picture of my farm. This picture represents that regardless of the situations/issues we need to be hopeful, eat well, avoid stresses. I had some financial issues because I lost my job during COVID, so planning has helped me to cope with the stress.”

“I figured out that this picture could signify my life (the dark period) when I was very sick, I took this picture. I see it being surrounded by lots of kind of difficulties and we can see that there is like dread and it’s still fighting so I really am I’m a fighter. I lost my job and worried about my debt and bills, and the mortgage but I am still very hopeful like the purple flower of this picture.” The adverse consequences of unemployment are further substantiated by the findings of [37,38]. Immigration provides an important benefit to Canada, increasing its economic growth and supporting diverse cultures [11]. Immigrants entering Canada are often healthier than other individuals entering Canada due to the strict criteria and screening an individual must pass to be approved for immigration [39]. While immigrants may experience better health when entering Canada, research has shown that their health deteriorates the longer they reside in Canada [39]. The vulnerability and honesty of the participants’ photovoice highlight the systemic oppression experience in Canada. Canada’s reliance on immigrant labor stands in stark contrast to the systemic inequalities that continue to marginalize vulnerable populations, particularly IRLHIV. Newcomers encounter barriers in immigration, healthcare, and employment [11].

This research reveals a pressing need for urgent and comprehensive action to address the social determinants of health that disproportionately affect this community. The COVID-19 pandemic has acted as a magnifying glass, exposing and intensifying the pre-existing inequities experienced by IRLHIV including the SDH of racism, discrimination, access to nutrition, healthcare, housing, and employment. This research, employing a mixed-methods approach that combined quantitative survey data with the deeply personal narratives captured through photovoice participatory action research, provides a sober glimpse into the lived experiences of IRLHIV in Alberta, Canada.

The COVID-19 pandemic is discussed as a “universal challenge”, a trauma globally felt. Yet the experiences of survival during the pandemic were not static. The data in Figure 2 clearly show how injustices and disparities in SDH were felt exponentially by IRLHIV prior to COVID-19 as well as how these challenges were compounded during COVID-19. This study exposes inequities and injustices experienced by populations already neglected by the government and social systems. To be effective, government policies and programs must recognize and understand the precariousness of housing, employment, food stability, and health for IRLHIV as well as develop robust social policies to address these disparities within SDH.

These findings serve as an urgent call to action. It is imperative that we move beyond simply acknowledging these inequities and take decisive steps to dismantle the systemic barriers that perpetuate them. This will require a multi-faceted approach that necessitates comprehensive yet nuanced social policies that focus on providing accessible, stable, and culturally appropriate support and services for IRLHIV. Service providers, academics, activists, and policy makers need to work collaboratively with IRLHIV communities to ensure their voices are heard to ensure that the very real challenges facing this population are being addressed in a culturally safe and sustainable way. In order to meet these goals, further community-based participatory action research is needed.

### 4.3. Study Limitations

This study is subject to several limitations. First, the use of snowball sampling can lead to the overrepresentation of certain groups. This was less of a concern for this study as the focus was on the lived experience of IRLHIV. However, snowball sampling relies on relationships between participants which can limit diversity within that group. This study was limited to Alberta, Canada, which has its own unique cultural, social, and economic characteristics. It also used a cross-sectional approach. These factors limit the generalizability of the study.

## 5. Conclusions

This community-based participatory study was designed with the aim to provide those who are working with IRLHIV in Alberta, Canada, an opportunity to understand intersectional oppression experienced by IRLHIV across Alberta, with a specific focus on pre-COVID-19 and during COVID-19 experiences. Through the innovative use of both a survey and photovoices, this mixed-methods study identified five key social determinants of health experienced by IRLHIV in Alberta, Canada: (1) racism and discrimination, (2) access to nutrition, (3) access to healthcare, (4) access to affordable housing, and (5) employment status. Quantitative and qualitative findings showed the pervasive and detrimental effects of racism as well as the barriers IRLHIV experience when accessing essential resources and opportunities. The use of mixed-methods allowed for a multifaceted understanding of the lived experiences of IRLHIV in Alberta, Canada, before and during COVID-19. By examining these lived experiences, this research provides valuable insights into the unique challenges faced by this population and offers recommendations for culturally appropriate supports, services, and policies to address health inequities faced by IRLHIV in Alberta. The findings from this study lay the foundation for further research into SDH inequities experienced by IRLHIV.

## Figures and Tables

**Table 1:** Sociodemographic characteristics of participants (n = 124).

Sociodemographic Characteristics	n	%
Gender identity		
Female	52	47.3
Male	55	50.0
Female-to-male transgender	3	2.7
Sexual orientation		
Straight/Heterosexual	77	68.8
2SLGBTQ+	35	31.3
Marital status		
Single	47	43.1
Married/partnered	44	40.4
Divorced/widowed	18	16.5
Ethnicity		
African	38	34.2
North American	20	18.0
South/Central American/Caribbean	10	9.0
Asian/Middle Eastern	15	13.5
Highest educational level		
High School and/or Lower	11	10.1
Trade, diploma, below Bachelor’s degree	46	42.2
Bachelor’s degree	41	37.6
Graduate degree (MSc, MBA, MD. PhD, etc.)	11	10.1
Living Situation	17	34
Live alone	30	27.3
Live with spouse or common-law	37	33.6
Live with family members or friends	43	39.1
Income (Fiscal Year 2022)		
Less than $9999	22	19.6
$10,000 to $24,999	24	21.4
$25,000 to 49,999	22	19.6
$50,000 to 74,999	22	19.6
$75,000 to 99,999	17	15.2
$100,000 to 149,999	3	2.7
$150,000 and greater	1	0.9

Descriptive and bi-variate analysis was used to analyze the data.

**Table 2:** Descriptive statistics and correlations for discrimination, stigma, healthcare services and social support.

Variables	n	m	SD	1	2	3	4	5	6
Discrimination	108	13.15	2.3						
2. Stigma	108	26.11	4.7	0.257 *					
3. Healthcare services during COVID	108	18.04	2.1	0.155	0.330 *				
4. Healthcare services before COVID	108	14.33	2.5	0.297 **	0.215 *	0.059			
5. Social support during COVID	108	18.75	3.3	−0.237 *	−0.225 *	0.032	0.022		
6. Social support before COVID	108	24.72	4.1	−0.249 *	−0.219 *	0.081	−0.045	0.122	

**—correlation is significant at the 0.01 level (2-tailed). *—correlation is significant at the 0.05 level (2-tailed).

## Data Availability

The datasets presented in this article are not readily available because of participant confidentiality. Requests to access the datasets should be directed to Rita Dhungel at rita.dhungel@ufv.ca.
